# How nurses in acute care experience professional pride: a qualitative study

**DOI:** 10.1016/j.ijnsa.2026.100624

**Published:** 2026-07-09

**Authors:** Johanna Ristau, Roman Helbig, Angelika Schley, Patrick Ristau, Corinna Peifer, Katrin Balzer

**Affiliations:** a Institute of Social Medicine and Epidemiology, Nursing Research Unit, Universität zu Lübeck, Germany; b University Hospital Schleswig-Holstein, Campus Lübeck, Germany; c Faculty of Applied Health Sciences, Deggendorf Institute of Technology, Germany; d Alsterdorf Assistenz Ost, Germany; e Institute of Psychology, Universität zu Lübeck, Germany

**Keywords:** Attitude of health personnel, Job satisfaction, Nursing, Nursing staff, hospital, Qualitative research, Working conditions

## Abstract

**Background:**

Professional pride is considered a positive emotion toward one's occupation. To date, there has been little research on professional pride among nurses, and the construct – including its defining characteristics, sources, and influencing factors – is still poorly understood**.**

**Objective:**

Our study aimed to explore how German nurses in acute care experience professional pride, including its sources and influencing factors, and the situations in which it arises.

**Methods:**

Qualitative interpretative study using data from semi-structured interviews and focus groups with a purposive sample of nurses working in a German university hospital and subsequent framework analysis.

**Results:**

We conducted 13 interviews with 17 nurses working in general wards, high-dependency units, and intensive care units at a German university hospital in May and June 2022. We found that the nurses experience both stable and situational professional pride and identified factors that promote and inhibit their feelings of pride. Individual pride sources include nurses’ attitudes towards the profession, their knowledge and competencies, and their care activities and successes. External factors that seem to influence whether these sources can be activated and sustained in actual practice include societal and organizational factors as well as workplace acknowledgement and appreciation. Especially nurses' implicit and explicit evaluations of care and treatment episodes seem to play a central role for their experience of professional pride.

**Conclusions:**

Based on our data, we developed a preliminary conceptional model of nurses’ professional pride as well as a working definition of this construct. Our findings can be used as a basis for future research which should focus on the transferability of the conceptual model to other settings and regions. Further research should also aim to develop psychometrically robust instruments to measure nurses’ professional pride and assess the effects of targeted interventions.

**Tweetable abstract:**

A qualitative study that provides a preliminary conceptual model of nurses' professional pride and proposes a working definition

**What is already known**

• Professional pride is considered a positive emotion that includes self-reflection or evaluation as well as a positive attitude towards one’s professional group.
• The professional pride of nurses has been little researched and there is still a lack of a standard definition or comprehensive descriptions of the phenomenon.
• While there are hints on aspects, influencing factors and consequences of nurses’ professional pride in the literature, focused research exploring and conceptualizing the phenomenon is still needed.

Alt-text: Unlabelled box dummy alt text

**What this paper adds**

• Based on interviews with 17 nurses from a German university hospital, we developed a preliminary conceptual model of nurses’ professional pride, distinguishing between situational and stable professional pride.
• The model integrates sources of nurses’ professional pride as well as external influencing factors and shows nurses' implicit and explicit evaluations of care and treatment episodes seem to be central to their experience of professional pride.
• Based on our findings, we propose a working definition of nurses’ professional pride that can be used as a basis for research.

Alt-text: Unlabelled box dummy alt text

## 1 Background

Pride is a self-conscious emotion that shapes human social behavior and group dynamics (Tracy et al., 2020). Authentic pride largely results from successes that are attributed to a person’s own effort and is associated with feelings of confidence, accomplishment, and self-worth (Weidman et al., 2016). Pride of an individual referring to their occupation is labeled "professional pride", which Hong et al. (2019) describe as "a positive emotion that includes self-reflection or evaluation and attitude toward one's occupational group".

In recent times, professional pride of nurses has become a popular motive of campaigns launched by healthcare services or nursing organizations to attract younger people to the nursing profession and to sensitize politicians and the public to the societal value of nurses and the need for sufficient working conditions (e.g. RNAO, 2023). In addition, slogans such as "Proud to be a nurse" have become commonly shared mottos on social media, and networks of nurses and universities post lists of reasons to be proud to be a nurse (The Network; Walden University, 2022).

However, despite this rise of popularity in public and political communication, nurses’ professional pride as a theoretical construct is still poorly understood. A systematic literature search conducted for the preparation of the current study revealed that the body of knowledge on pride in nursing is very limited (see below and Supplement 1). Additionally, to date, neither a generally accepted definition of professional pride in nursing nor a comprehensive description of its determinants and influencing factors exist (Adlhoch, 2024a; Quernheim and Zegelin, 2022).

### 1.1 Professional pride: conceptional considerations

In nursing literature, professional pride is often positioned alongside other constructs such as professional identity, self-image, self-concept or work satisfaction. For example, Öhlén and Segesten (1998) mention professional pride in their concept analysis on nurses’ professional identity, stating that professional identity is a prerequisite for nurses to develop a realistic professional self-image and to feel professional pride. In their analysis, they define nurses’ professional identity as the person's feeling and experience of her/himself as a nurse and other people's image of the person as a nurse as well as being interwoven with the nurses personal identity. Fagermoen (1997) takes another perspective, describing professional identity more as a value-based guiding principle that shapes nurse's actions: „Professional identity refers to the nurse’s conception of what it means to be and act as a nurse; that is, it represents her/his philosophy of nursing. (…) More precisely, professional identity is defined as the values and beliefs held by the nurse that guide her/his thinking, actions and interaction with the patient.“ Besides these, there is a wealth of definitions for professional identity in nursing and health professions and different identity theories are used as a basis, depending on the research interest (Cornett et al., 2023; Fitzgerald, 2020). However, contrary to pride, *professional identity* is not used to describe an emotion.

Quernheim and Zegelin (2022) describe nurses' professional pride as a sense of pride that is grounded in knowledge gained through professional training and a sense of security, and that relates to performing their profession, i.e. working as a nurse. They differentiate it from mere job satisfaction by describing that the nurses do not only perceive the work situation itself as positive but consider their personal performance – by its nature and quality – to adding value to the provision of care in their facilities. Consequently, they describe nurses’ professional pride as an attitude based on cognitive beliefs and accompanied by positive emotions. Furthermore, they state that nurses’ professional pride includes their achievements and encompasses “a strong sense of self-respect, combined with public recognition of nursing work”, and outline six aspects of professional pride, including identity: (1) knowledge and education, (2) identity and individuality, (3) courage and motivation, (4) meaningfulness, (5) passion, and (6) self-esteem. However, they do not describe how these aspects have been identified and how they understand them. The same holds for the first scale to measure nurses' professional pride, the Nursing Professional Pride Scale (Jeon et al., 2020), which has also been translated into Turkish (Aydin et al., 2022). It consists of 27 items in 5 categories – feeling of vocation, role satisfaction, role of problem solver, self-achievement, and willingness to stay – but its theoretical foundation remains unclear.

A construct similar to professional pride that has been studied in Germany is work pride (“Arbeitsstolz”) in nursing, as described by Fuchs-Frohnhofen and Bessin (2019). The authors deduce work pride from manufacturer pride – an emotional outcome of an evaluation process in which one’s own work is regarded as a success because it meets or exceeds one’s own expectations – but state that the work product in the service sector and especially in nursing are immaterial, less visible and measurable, and rather short-lived. In contrast to professional pride in nursing, as described above, the concept of work pride does not appear to encompass values, beliefs, or a sense of meaning for the individual, but rather focuses on the evaluation of processes, outcomes, or one’s professional standing.

Krämer and Rhein (2012) also discuss pride as a positive emotion experienced when one meets – and especially exceeds – expectations regarding one’s own work performance. They use the term ‘performance pride’ and note that the moments in which this type of pride is experienced are often short-lived because they are tied to specific tasks. Therefore, performance pride can only represent a part of the more complex and, by comparison, more stable construct of professional pride.

In summary, it can be stated that professional pride is not simply equivalent to professional identity or job satisfaction, but rather appears to be a positive, self-evaluative emotion that is connected with professional identity and professional competence. Furthermore, it encompasses a self-appraisal of the nurses’ performance and achievements through delivering their nursing care.

### 1.2 Literature review

To gain an initial understanding of how the construct “professional pride” is used in nursing literature, we searched MEDLINE via PubMed and CINAHL for publications touching on the professional pride of nurses working in somatic acute care or long-term care facilities. Additionally, we carried out selective hand searches. After removing duplicates, we screened 696 publications and finally extracted findings on nurses’ professional pride from 17 studies (11 from Europe, the others from the USA, New Zealand, South Africa, and Iran). A detailed overview of retrieved studies and their main findings is provided in Supplement 1.

Most studies addressed nurses' professional pride only in passing. The majority of findings relates to single statements in qualitative studies, e.g. in connection to categories that included nurses' comments on the quality of their work, their competencies and their perception of the nursing profession, or as a factor that positively affected their retention (Chegini et al., 2019; Hølge-Hazelton und Berthelsen, 2021; Roth et al., 2022). Only in five studies, addressing nurses’ pride was planned a priori: The study of Chegini et al. (2019), which focused on the relationship between occupational stress, work-related quality of life, and turnover intention among intensive care nurses, contained one pride-related item in a questionnaire, namely on “job pride”, which was defined as “feeling pleasure and satisfaction with your job and in the organization”. They found job pride to be a protective factor regarding ambitions to change profession. Nydahl et al. (2015,^2016^) studied the experience and extent of work pride (as described by Fuchs-Frohnhofen and Bessin, 2019) and recognition in intensive care nurses. However, likely due to the phenomenon under study, the nurses’ descriptions of pride mainly referred to specific work experiences, such as collaboration in the team or acting in critical situations, but not to aspects like the personal meaning of the nursing profession for the nurse or consequences of their pride. The other two studies were not conducted in the acute care sector. Sneltvedt and Bondas (2016) examined the experience of professional pride among newly graduated nurses (i.e. nurses who have a higher level of education than their colleagues and therefore take on a more responsible role) in inpatient long-term care or outpatient care. They describe professional pride in nursing as a “complex phenomenon with relational, dynamic, and collective dimensions”, identifying the relationship with patients as an important source of pride. The study of Vikström and Johansson (2019) focused on nursing home staff's experiences of how a quality development project influenced their work. They considered professional pride “a strong sense of pride in specific work‐related competencies” and found that nurses were proud of their experience and competence gained through direct care activities and that they took pride in doing good work for the benefit of residents.

Overall, these findings support the above notion that professional pride is a construct that intersects with nurses’ professional identities but especially arises from a positive self-evaluation of their knowledge, competencies and performance. However, a generally accepted definition and a comprehensive, empirically grounded description of the construct, including its determinants and influencing factors, are still lacking. More research is required to disentangle and describe the defining concepts, such as noticeable characteristics, influencing factors and potential outcomes of nurses' professional pride.

Therefore, our study aimed to explore how nurses in an acute care hospital experience professional pride. Additionally, we investigated the sources of nurses' professional pride, its influencing factors, and the situations in which it arises. The following research question guided this qualitative study:

*How do nurses at a German university hospital experience the phenomenon ‘professional pride’?*

## 2 Methods

### 2.1 Design

A qualitative approach following the interpretative paradigm was used to explore nurses’ subjective views and gain a better understanding of professional pride in nursing. We conducted semi-structured interviews and focus groups with nurses working in acute care and analyzed them by applying framework analysis (Ritchie et al., 2014).

### 2.2 Context

This study took place in a university hospital in Germany. In this country, the possibility of primary academic education in nursing has only existed for a few years. Consequently, almost exclusively vocationally trained nurses work in this profession. The proportion of academically qualified nurses involved in bedside care in university hospitals still stagnates at around 2% (Bergjan et al., 2021). Professional registration of nurses is not mandatory, and there are no nationwide structures of professional self-governance. Therefore, in this study, the term “nurse” refers to all nurses who completed a minimum of three-year education in nursing, whether through vocational or university undergraduate programs. In addition, nurse-to-patient ratios in Germany differ from those internationally: On average, one nurse in a hospital cares for ten patients, significantly more than in many other countries (Rafferty et al., 2019), whereas in intensive care units it is usually two patients per nurse (Jorch et al., 2010).

### 2.3 Setting and participants

Nurses were approached via a printed study invitation on their ward and via electronic information forwarded by their team leaders. Table 1 shows the eligibility criteria. We invited nurses from different wards and ICUs to participate in the study (e.g. gynecology, cardiology, internal medicine, general and specialized surgery). Additionally, we purposively included nurses with varying levels of experience and expertise. Some nurses were approached directly by author 1 to increase variation in the sample, e.g., because they held a bachelor's degree in nursing or had already resigned to leave the profession due to job dissatisfaction. Recruitment was stopped when 17 nurses from 6 wards with different characteristics participated in the study and no other nurses chose to participate within the fixed time frame designated for data collection. Five additional nurses who had been willing to participate in the study in the first place had to withdraw before the interview due to time constraints or sickness.

**Table 1:** In- and exclusion criteria.

Inclusion criteria	Exclusion criteria
Nurses • with at least one year of experience after receiving their state occupational permit • working in general wards, high-dependency units, and intensive care units of various disciplines (medical, surgical, and mixed)	Nurses working in mental health units, pediatric wards, or operating rooms (due to the very divergent, highly specialised tasks and working environments in patient care)

### 2.4 Data collection

Initially, data collection was planned exclusively via focus groups. We aimed for five 2-hour focus groups at predetermined times. Due to recruitment challenges, we also offered individual interviews at the nurses’ convenience. Prior to data collection, a topic guide was developed based on findings from the literature and the research questions (identical for focus groups and interviews), containing items on the nurses' feelings of professional pride, their understanding of the phenomenon, situations in which they feel proud, and factors influencing their pride (Supplement 2). To pre-test the topic guide regarding comprehensibility, length and the potential of the questions to evoke comprehensive statements, we tested it in a pilot focus group with six newly graduated nurses (nursing students after their professional registration and working part-time as a nurse). This pilot focus group was neither audio-recorded nor analyzed. Feedback from the pilot interviewees revealed no need for changes in the topic guide; all questions were easily understood and evoked comprehensive responses. The guide was then used flexibly in the interviews and could be supplemented with spontaneous questions. There was no need for alterations during data collection.

We planned to conduct all focus groups on university premises with moderation by author 1 and a colleague taking notes. For individual interviews, the nurses could choose the location and decide whether the interview should be conducted in person, via a video platform (Cisco Webex, Cisco, licensed by the university), or by telephone. Author 1 conducted all focus groups and interviews; they were audio-recorded and transcribed verbatim (including capitalization of stressed words, slashes for fractions of sentences or words, and dots for pauses). After comparison with the recordings, transcripts were anonymized and imported into MAXQDA 2022 (VERBI).

Before each interview, nurses completed a questionnaire on demographics, other characteristics (e.g., length of professional experience and additional qualifications), and two validated scales to detect variance in job-related characteristics (Professional Self-Efficacy Scale (BSW-5-Rev) (Knispel et al., 2021), and the Identification with Occupation and Activity subscale of the COBB (Commitment Organization, Occupation, and Form of Employment) (Felfe et al., 2014).

### 2.5 Data analysis

Sample characteristics and standardized scores were descriptively analyzed with Microsoft Excel 365 (Microsoft Corporation) and IBM SPSS Statistics (IBM) by author 1 with support of author 4.

For qualitative analysis, we used the framework analysis method, an approach intersecting with thematic analysis (Braun and Clarke, 2006) and aiming to condense highly structured results from summarized data through a straightforward, step-by-step process (Ritchie et al., 2014). Author 1 read through the first three interview transcripts several times, noted topics and subjects of interest, and then inductively developed an initial thematic framework for the subsequent categorization of interview excerpts. Author 3 also read the transcripts and discussed the framework with author 1. Discussions also occurred with authors 5 and 6, the study's supervisors. After agreement on the themes in the framework, authors 1 and 3 independently coded the first three interviews using the initial framework. However, compared to other qualitative research methods, “coding” in framework analysis describes rather a sorting of data, indexing them to pre-specified themes. In our study, the main themes used for coding were: (1) positive attitude toward the profession, (2) professional identity, (3) competence, (4) appreciation of the profession and one's work, (5) working as a team, and (6) stressors/challenges (each with subcategories). Table 2 shows the examples for the categories and subcategories in themes 1 and 2.

**Table 2:** Coding scheme for themes 1 and 2.

Theme 1: positive attitude toward the profession	Theme 2: professional identity
• Professional pride • Level of pride • Description/definition of professional pride • Situations in which pride is felt • Situations in which no pride is felt • Explicitly stated reasons for pride • Explicitly stated barriers for pride • Effects of pride • Enjoyment of one's work • Explicit statements on fun/enjoyment (positive) • Explicit statements on fun/enjoyment (negative)	• Conception of the nursing profession • The importance of the profession for the nurse • Role and responsibilities of a nurse • (Required) attitudes/characteristics of a nurse • Key elements/approaches of professional nursing care • Objectives of nursing care • Patient-centered care • Spiritual and emotional support • (Un)successful provision of care/treatment • Taking on responsibility • Quality

The codings, i.e. the assignments of the nurses’ interview statements to the themes, were very similar between authors 1 and 3, meaning that both researchers assigned the same statements to the same theme. Slight discrepancies, e.g. regarding the length of the coded statements, were discussed in an online meeting, resulting in agreement and further clarification on the coding process, with no need to adapt the framework as such. Consequently, the framework was deemed suitable for indexing all interviews and was subsequently applied by author 1, who coded all remaining interviews by assigning the suitable statements from the interviews to the respective themes in the framework.

All coded segments per interview and theme were then summarized by author 1. Summaries of themes that focused mainly on the phenomenon of interest – i.e., study participants' definitions of professional pride in nursing, specific situations when they felt or didn't feel pride at work, and factors influencing their pride – were then coded inductively, i.e. they were rewritten in a shortened form, but staying close to the original text. In framework analysis, these codes are called “detected elements”. Table 3 illustrates the coding on an example.

**Table 3:** Example of coding a summary.

Interview	Summary	Detected elements
**I11**	Working in nursing is very important. She can help people, be herself, communicate well with patients, and listen to their needs, which allows her to do her job well. Proud because she can identify problems and find solutions, which leads to an improvement in the patients’ situation. Proud of her expertise, but she can still improve it. Proud of working at the bedside, providing care, and being by her patients’ side, not on the computer.	• Doing meaningful work • Being able to help people • Being able to be yourself at work • Being able to identify patients’ needs through communication • Being able to listen to patients (sometimes that’s all they need) • Proud of expertise • Expertise can always be expanded • Proud of bedside nursing, because being with the patient is more important than being on the computer

All detected elements were then grouped, sorted, and provided with initial, partly interpretative headings (e.g., "Feeling good about the work you have done") using a separate text document. These grouped elements were then reorganized according to similarities and merged into more abstract categories (e.g., "Positive evaluation of the care situation"). These steps of the analysis were conducted by author 1 and author 2. Summaries on other themes were consulted later in the analysis to gain deeper insights into specific aspects of pride-related factors (e.g., on the relevance of specialist competencies and expertise or ethical issues).

Supplement 3 contains an example of grouped statements. Subcategories such as “Positively influence the patient's state and condition” and “Achieve visible/ tangible results through nursing care” were grouped into the category “Making a positive difference through your own contribution”.

Afterwards, the essence of the categories and identified interrelations among them were visually summarized in preliminary models on sources of nurses’ professional pride, factors that negatively influence feelings of pride, and the evaluation of care and treatment episodes (Supplement 4). These preliminary models were developed by authors 1 and 2 and additionally discussed with author 4. Later, they were combined and further developed into an overarching exploratory conceptual model (authors 1, 2, 4 and 6).

For example, the above-mentioned category “Making a positive difference through your own contribution” is found in the preliminary model of the nurses’ sources of pride, named as “the nurse’s achievements and successes” and labeled as a “self-related – individual” source of pride (Supplement 4). In the final exploratory conceptual model, it is found as the pride source “The nurses‘ care activities and successes” (see Results section).

In addition, at the stage of the development of the first visualizations, author 1 consulted with several nurses from the sample regarding the findings to verify whether they were coherent and complete from the participants' perspectives. The nurses' feedback confirmed the analysis and contributed to a better understanding of the data.

### 2.6 Researcher characteristics and reflexivity

Six researchers conducted this study: four nurses with acute care experience, one paramedic and health scientist, and one psychologist. Author 1 works mainly in direct patient care, author 2 in nursing management as well as academia, and authors 3 to 6 in academia. All co-authors are experienced in qualitative research. Authors 5 and 6 supervised the planning and implementation of this study.

Author 1, who played a leading role in the development and implementation of the study, including the analysis and reporting of the results, is a nurse with a bachelor's degree in nursing science. Due to her work in intensive care and the literature review, she was sensitized to possible ward-specific differences and factors that may influence nurses' professional pride. Furthermore, she knew some of the participants before the study. Throughout the study, she constantly made herself aware of her preconceptions and used purposeful inquiries in the interviews rather than assuming to know what the nurses meant. She reflected on each interview through memo-writing, including her (perceived) role and behavior as an interviewer, and captured first analytical ideas. Repeated discussions among all authors, additional experienced researchers, and consultation with study participants contributed to constant reflection throughout the analysis.

### 2.7 Ethical considerations

Before recruitment was started, the Ethics Committee of the Universität zu Lübeck provided ethical clearance (file 22–166). Study participation was voluntary. All nurses gave written and ongoing informed consent to participate in the study and audio recordings of the interviews. Withdrawal from the study was possible at any time before and during the interview without providing reasons or negative consequences. Five nurses chose to withdraw before the interview due to time constraints or illness.

We conducted all data analyses using anonymized data and complied with the General Data Protection Regulation (GDPR).

## 3 Results

### 3.1 Sample

We conducted a total of 13 interviews (abbreviated with “I” and a number, with lower number indicating earlier interviews in the data collection timeframe) with 17 nurses in May and June 2022. Besides 11 individual interviews, there was 1 duo interview (I7) and 1 focus group with four nurses (I5). 8 interviews were conducted on university or hospital premises, 3 via video conference, and 2 by phone. All interviews were held by author 1, with support of author 3 in the focus group. Interview duration ranged from 37 min (Interview 13 [I13; with lower number indicating earlier interviews in the data collection timeframe]) to 2:01 h, with a median duration of 58 min. Total time for interviews was 13:49 h.

Nurses' characteristics are presented in Table 4. Although most of the nurses were similar in terms of having more than ten years of professional experience, they contributed diverse perspectives on the research topic due to the differences of the wards where they worked, their individual shift preferences, and their additional qualifications. Furthermore, even though they all work at the same university hospital, they also have experience from other hospitals, e.g., from previous employments or because they are currently also working as temporary staff.

**Table 4:** Sample characteristics.

		*N* of nurses (percent)
**Gender**	Female	11 (65)
**Age group**	20–29 years	2 (12)
	30–39 years	4 (24)
	40–49 years	7 (41)
	50–59 years	3 (18)
	≥ 60 years	1 (6)
**Nursing education**	Vocational training in Germany	13 (76)
	Academic degree	4 (24)
	Professional license obtained in a country other than Germany	3 (18)
**Length of professional experience as a nurse**	1–2 years	1 (6)
	3–5 years	1 (6)
	6–10 years	1 (6)
	> 10 years	14 (82)
**Job size**	Part-time (> 50% of a full-time equivalent)	5 (29)
	Full time	12 (71)
**Ward characteristics**	High dependency or intensive care unit	11 (65)
	General ward	6 (35)
**Additional qualification or specialized training**	Oncology care	1 (6)
	Intensive care	7 (41)
	Wound care	1 (6)
	Nurse preceptor	3 (18)
	Nursing team leader	6 (35)
	Other*	6 (35)

⁎ Other qualifications included: specialist nurse dialysis and nephrology, quality management specialist, respiratory therapist, ethics consultant, pain nurse, barrier nurse, nurse specialist for movement promotion and repositioning.

In the BSW-5-Rev, nurses scored an average of 3.4 (standard deviation (SD) 0.4; median 3.4, range 2.6–3.8), indicating high to very high professional self-efficacy. Among COBB’s professional commitment subscales, the mean value was highest for affective commitment (COBB BCA, mean 4.1, SD 0.9; median 4.4, range 2.0–5.0).

### 3.2 Levels of professional pride in the sample: scale values vs. interview data

The COBB scale contains an item specifically focusing on pride ("I am proud to work in this profession"). The mean value for the ordinal-scale item was 5 (mode 5, range 2–5), indicating a strong feeling of professional pride in the sample.

While some nurses also expressed high levels of professional pride in the interviews, others provided a more nuanced view of their professional pride and some revealed conflicting feelings. Answers on the first question in the interview (“In what ways are you proud to work as a nurse?”) show that some nurses find it complex and challenging to rate how proud they feel:

*I have to admit, I find it hard to answer that question – whether I’m proud. I think I used to be proud back in the days, but nowadays I can’t really say I am. So… do I have to answer “yes”? (I3)*

*My view of being proud is VERY complicated. I chose this career because I like it, and in principle, I still do like my job. But I have to be completely honest: for at least twenty years, I’ve been telling anyone who asks me if they should become a nurse, “Don’t do it – find something else.” Because the circumstances have just changed so much. (I5, nurse 2)*

*I find that a really tough question, because I actually enjoy what I do. (…) But it’s just hard to say how or why I’m proud of it. Because sometimes the circumstances just don’t allow you to be proud. (I5, nurse 3)*

*I am proud/ Being a nurse is what I’ve always wanted to do. But I find it hard to say whether I’m still really proud to do it. I used to be, yes, but these days the job has nothing to do with what I learned back then, and so/ Is anyone still that proud these days? It’s hard to say. I don’t think it has much to do with pride. (I9)*

*I find it hard to answer this question, because I’ve never really thought about it before – whether I’m proud or not. I’d say “yes,” I am proud. But it’s not like I feel a lot of pride in certain situations or every day when I go to work. (I10)*

These quotes already show that nurses view professional pride as a complex phenomenon that they associate both with the profession itself and with their actual work in the hospital.

### 3.3 Nurses' understanding of “professional pride”

The nurses in our study described professional pride as feeling proud to be a nurse. Some of them stated that being a nurse belongs to their personality (I6) or that working as a nurse gives them a way to express their personality *(“I can help a lot of people, and I can be exactly who I am”*; I11). They stated that professional pride goes beyond job satisfaction (“*Pride is a little more [laughs] than just being satisfied. It's kind of an overall package*”; I7), meaning they were happy and in harmony with the profession.

### 3.4 Dimensions: situational and stable professional pride

When asking the nurses in our study about situations when they experience professional pride, many nurses reported feeling proud when they use their skills, experience to make a difference for patients, and provide care that meets their professional standards and values. In terms of time, they feel pride during or after direct care activities, as well as after longer care and treatment episodes (Table 5). The last one was mostly mentioned by intensive care nurses; they reported on critically ill patients they "had already given up on" or they had "really fought" for (I5), for whom they were able to achieve significant health improvements as a team. In retrospect, they evaluated that what they did was right and the effort had been worthwhile. Overall, a subsequent reflection on the care seems to be an important element of nursing work, with many nurses in the sample reporting to reflect on their shift and their achievements while on the way home.

**Table 5:** Subcategories of situational professional pride.

Time of occurrence	*Pride experienced in a specific situation*	*Pride experienced after a situation or workday*	*Pride experienced looking back on longer care episodes*
**Description**	Successes such as using newly acquired skills or the application of caring behaviors	Nurses' feelings of pride when going home, reflecting on their performance and the effect of their work during their shift	Feeling proud when reflecting on individual or team achievements in the context of longer care episodes, sometimes including effort-benefit comparisons
**Examples from data**	*I feel proud when I do something and the patient benefits. (I1)* Nurse I4 gives examples: a properly applied wound dressing; a palliative situation in which one just holds someone's hand; having time and sitting with a patient Nurse I1 reports on a situation: noticed that an agitated patient's ventilator filter was blocked with fluid; after changing the filter, the patient no longer needed sedation and was successfully extubated instead of tracheotomized Nurse I11 reports on a situation: noticed that a covered-up drainage tube was twisted, thus fixed it with a new dressing; the patient was breathing better shortly after and no longer needed supplemental oxygen	*I am going home, and my patients are doing well. Something has improved. On that day, I can say: I made a difference because I was here. I feel good for myself. (I5)* *When I go home, I know I have done something good for someone, which makes me proud. (I1)* *I am proud when I have completed my workday and have been able to care for a patient well,* i.e.*, holistically. (I4)*	*I am proud to help patients to improve certain things. For example, when you notice in wound care that something is developing for the better. (I12)* *And the colleague just used a lotion [with incontinence-associated dermatitis] that didn't help at all, so I said, "We won't use that anymore. We'll use something else." So I started using a different lotion, and it helped a lot. (I11)* Nurse I13 and a nurse in I5 reports on critically ill patients the nurses *"had already given up on"* or they had *"really fought"* for, for whom they were able to achieve significant health improvements as a team; in hindsight, evaluating that they did the right things and the effort had been worthwhile

In contrast to these descriptions of situational pride, some nurses rejected the idea of feeling professional pride in specific situations:

*I just really enjoy my job. There are no situations in which I feel a particularly pronounced professional pride. I do what I do, and I really enjoy doing it. (…) But any particular professional pride? Well, I think it's actually always there. (I8)*

*Yes, I do feel proud. But it's not that I feel real pride in certain situations or every day when I go to work. (I10)*

They stressed that their pride was rather independent from specific situations and that they experience it more subtly (*“background noise”*, I8). This led us to distinguish between situational and stable professional pride, with stable pride being what the nurses described as a personal intrinsic characteristic and as an emotion that was meaningful to them, and situational pride as a spontaneous, context-dependent positive feeling.

When analyzing the reasons for their pride as reported by the nurses in our sample, we found that most nurses in our study experience both situational and stable professional pride. Furthermore, our data suggest that experiencing situational pride regularly might play a role in keeping up or strengthening nurses’ stable professional pride, and that there are certain influencing factors that appear to have an impact on whether nurses can maintain their stable professional pride.

### 3.5 Key findings on nurses' professional pride: sources, influencing factors, and possible effects

**Fig. 1:**
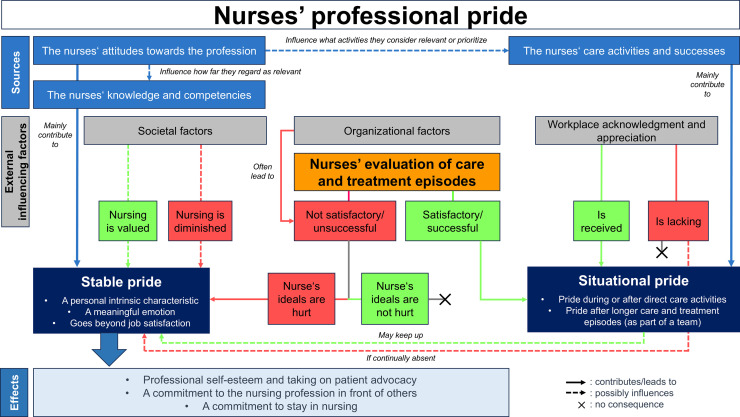
Exploratory conceptual model summarizing nurses’ professional pride.

In the interviews with the nurses, we identified three sources of professional pride: nurses’ attitudes towards the profession and their individual knowledge and competencies, both of which seem to mainly contribute to stable professional pride; and nurses’ care activities and successes, which mainly seem to contribute to situational pride. While their professional attitudes, knowledge, competence, and care experiences constitute important individual-level sources of their professional pride, organizational and societal factors as well as the level of acknowledgment and recognition they experience in their workplace, e.g. their hospital, seem to influence whether these sources can be activated and sustained in actual practice. Particularly organizational factors and workplace interactions seem to influence how the nurses in our sample carry out and perceive daily patient care. How the nurses evaluate care and treatment episodes – i.e., whether they rate them as satisfactory or unsatisfactory – could be identified as central to their experience of professional pride. Finally, our data hint at possible effects of nurses’ professional pride. These included having professional self-esteem and taking on patient advocacy, committing to the nursing profession in front of others and a self-commitment to stay in nursing. Fig. 1 summarizes those findings in an explanatory conceptional model.

### 3.6 Sources of nurses' professional pride

In the following, we describe the professional attitudes, knowledge and competencies as well as care activities und successes that nurses referred to as sources of their professional pride in the interviews. These sources of professional pride are closely linked: The nurses' attitudes towards their profession can influence the value they place on expertise and what they see as relevant competencies, as well as what activities they prioritize and what they consider a care success.

**Attitudes towards nursing**: Nurses reported a predominantly positive attitude towards the profession, making them proud to be nurses – even though they are often frustrated by their work conditions. Some stated they enjoy working as nurses and could not imagine pursuing another career. Meanwhile, other nurses who had been in the job for many years claimed they no longer enjoyed it due to unsatisfactory conditions and a perceived deterioration of the care quality. However, they reported that they had previously liked working as a nurse very much and were fond of the profession how it was practiced a few years ago.

Nurses reported a predominantly positive attitude towards the profession, making them proud to be nurses. Some stated they enjoy working as nurses and could not imagine pursuing another career. However, some nurses reported ambivalent feelings: While they generally viewed the nursing profession as something that made them proud, they stated that working conditions and society’s perception of the profession had led them to no longer feel that pride. Especially nurses who had been in the job for more than twenty years claimed that they no longer enjoy working as a nurse, given today's approach to patient care in hospitals, which they linked to a decline in the quality of nursing care. Before, they had liked working as a nurse very much, and they were fond of the profession how it was practiced a few years ago.

The nurses in our sample described nursing generally as challenging and exciting, and appreciated the broad spectrum of professional activities, including interprofessional collaboration. They consider the profession as significant and meaningful at both a personal and a societal level, perceive it as a "sovereign, ethical task" (I4) and, overall, indispensable (*"Without us, there is no health"*, I2). This makes the nurses proud. Some nurses described nursing as a calling or a profession that not just anyone can take up and for which a high level of intrinsic motivation and particular character traits or "blessings" are necessary:

*You have to have the INTEREST in it. Some people are not suitable; they do NOT have the talent to commit to the patient or the job. (I3)*

*First of all, it's important that you like doing it, the job. (…) Anyone can learn anything, but you really have to want (…) this profession. (I8)*

*Knowledge and, of course, empathy and team skills: Not everyone can do that. Let's not kid ourselves, not everyone is given that. So, being in nursing is somehow also a bit of a calling. (…) You can't learn every capability. (I7)*

This perception of the prerequisites for working as a nurse − good, unique character traits that can be learned only to a limited extent − once again explains why nurses feel proud to be able to work in this profession themselves.

**Nurses' knowledge and competencies:** The nurses stressed their pride in their knowledge and competencies. They described specialist knowledge as a constitutive aspect of the nursing profession, which they regard as essential for ensuring patient safety, and emphasized that this knowledge must always be kept up to date. They highlighted the importance of medical knowledge, which they consider the basis for working at eye level with physicians, and sometimes compared themselves with others: ICU nurses explained that they had deliberately chosen this setting because they needed "more specialist knowledge" than in a general ward and that they are proud of being able to support their ward colleagues, "who do not have this BIG expertise, but rather other qualities in their professional focus", in emergencies (I7). They pointed out that they had invested significant effort in acquiring their competencies and that long-experienced intensive care nurses had more expertise than "the average doctor fresh to the ICU" (I5). They consider understanding physiological processes or the modes of action of drugs and knowing how to work with complex medical technology, which in some cases earned them "awe" (I7) from their environment, as central components of their expertise. They linked their knowledge to the ability to secure and save the lives of patients and reported that this significantly contributes to their pride:

*You have to realize, and I think people out there know this too, that WE know how to manage a ventilator, which DIRECTLY secures the patient's life. (I7)*

*I can save lives; that's the most important thing. When you've done your job one hundred percent, you go home with great pride, so it can't get any better. (I2)*

In contrast, nursing expertise not directly related to medical knowledge or skills was mentioned only casually (*"I know how to/or I think I know how to treat patients well. And not only in medical terms"*, I6), independent of the ward characteristics. Nurses described nursing knowledge as very comprehensive, but they were not able to specify it in more detail and often related it to prophylaxis.

*I am proud to be a nurse, which means that I can help my patients to get out of the difficult and potentially life-threatening situation they have gotten into and to get back to normal, and that help can take many forms. (I6)*

*"Expertise" actually covers a great deal in all areas, be it personal hygiene, prevention, or prophylaxis; all of that is somehow included. (I10)*

No nurses mentioned up-to-date scientific knowledge enabling evidence-based nursing practice as a source of pride. Instead, they emphasized the importance of experience, which they partly equated with expertise ("I have a lot of expertise through all my work experience," I8). When asked about expertise, they simultaneously stressed the importance of empathy and humanity.

*Yes, you need expertise. But you also have to be a good person. I think it's not enough to have good nursing or medical knowledge. (I1)*

*So, expertise is essential for me, but empathy is also crucial. I wouldn't say that one is more important than the other. (I10)*

There seem to be two dimensions to the importance of expertise: While some nurses − especially ICU nurses – consider (medical) expertise the essential element in their professional thinking and actions, others mention expertise as a crucial factor as well, but one that merely complements a foundation of other qualities such as empathy, certain "given" character traits, and interest in the nursing profession.

While having knowledge and competencies mainly contribute to nurses’ stable professional pride, successfully using this expertise in patient care also leads to situational pride.

**Nursing activities and successes:** Nurses' attitudes towards relevant knowledge and skills are closely related to specific descriptions of nursing activities and experienced successes, which represent the third source of pride we identified in our study. Nurses take pride in direct patient care and sometimes even place bedside care at the center of their professional pride. They view direct care as the core of nursing and an opportunity to use their self therapeutically for the patients' benefit:

*Working at the bedside is exactly what DEFINES our profession. And that is exactly what is BEAUTIFUL: Communicating with ill people, contributing to the treatment and recovery process, and giving patients something POSITIVE, a bit of strength and the experience you have – that is actually what is beautiful. And that is what makes me proud: If I can accomplish that, I am proud, and that's what I like to do. (I3)*

When providing direct care, the nurses in our sample stated to place great importance on patient-centered, individually planned care that builds on patients’ resources. They focus not only on physiological or medical but also on emotional and spiritual needs, with nursing activities including carrying out assessments, assisting with personal hygiene or in physiological procedures, and caring activities such as comforting and listening to patients.

Based on their philosophy of nursing and the characteristics of the wards they work in, the nurses named various successes and activities that contribute to their professional pride. Especially nurses working in an ICU defined success as saving patients’ lives and achieving measurable and visible improvements in vital signs or health status, including the discharge from the ICU to a general ward and the return to their normal lives. Other examples of nursing interventions that made the nurses in our sample proud included carrying out activities that promote patients' well-being and address their individual needs. Especially nurses working in general wards described care as successful when they could support or motivate their patients through caring behaviors, when they could give them emotional comfort and when they were able to help them with something. Against this background, patient mobilization plays an important role both as a patient-centered care activity and as an indicator of improvements in physical and overall function. Nurses rate mobilization as very relevant to patients in various ways and consider their contribution to mobilization improvements to be high:

*I have a patient in mind who was very difficult to mobilize, and you gave him input and instructed him how it could go better. And then you come back two days later and see the success, how he can almost do it independently. Then, of course, you are proud that you were a part of it and that he is happy that he can do it on his own. That makes me incredibly proud. (I7)*

*I'm the kind of person who likes to get patients out of bed, even if they have been admitted from a nursing home and can't do much anymore. (…) And I'm proud when people who have been in bed for, let's say, ten years are able to sit at the table and eat. (I6)*

Chronic wounds are another example of success in which nurses experience high self-efficacy, with nurses being proud to contribute to wound healing through their expert wound care.

Intensive care nurses further emphasized medical successes that they achieve through their expertise-based actions as a source of their pride and attribute these successes predominantly to themselves and their colleagues rather than to physicians:

*I am happy about almost every blood gas analysis that I improve. Especially because I think that many physicians are NOT able to do this and don't even recognize how important this is. So, playing around with the ventilator and truly seeing the success makes me proud. (I5)*

*I remember a few patients (…) who had already been given up on, but we still managed to get them through as a team. And patients in whom intubation or reintubation could be avoided through the individual performance of, let's say, two or three shifts in a row. So, I'm very proud of that. (…) Or when you have taken over a critical patient and ensured with your work that he is no longer critical by the end of the shift. (I13)*

Regarding needs-oriented, individual patient care, the nurses reported to feel proud when they can provide care that contributes to the patient's well-being. This includes an orientation of personal hygiene to the patients' wishes, for example, the use of their own grooming products, and sufficient time for individually planned nursing activities − even if this means deviating from the usual ward routine. They are also proud when they can successfully use their relationship with patients in specific communication activities to convince their patients of advancements in their treatment, to give them strength and hope for the further course of therapy, to get them "out of a depressive spot" (I6), or to rescue them from individual crises.

*A meaningful conversation with the patient also makes me happy if I can convince him that this is a good situation for him now, that we are quite happy with how things are going. (…) If the patient then feels more comfortable and at ease, that makes me proud. If I can convince him that everything is exactly the way it should be. (I7)*

Also, the nurses we interviewed expressed feelings of pride in the context of end-of-life care situations when they could sit at the patient's bedside and hold the patient's hand.

### 3.7 External influencing factors

While the nurses' professional attitudes, knowledge and competencies as well as their performance of direct nursing care and the successes they achieve by it constitute important sources of professional pride on an individual level, there are external factors that seem to influence whether or to what extent these sources can be activated so that the nurses actually feel proud. In our study, we identified societal factors, organizational factors, and receiving or being denied recognition and appreciation from others at the workplace to play a role. While workplace acknowledgement and recognition seemed to relate more to their experience of situational pride, societal perceptions and the public image of the nursing profession appeared to have the potential to influence the nurses’ stable professional pride. Organizational factors seemed to have a major influence on an element which we discovered to play a central role in the nurses’ experience of professional pride: their evaluation of care and treatment episodes.

**Societal factors:** The role of the societal image of nursing in nurses' professional pride was addressed primarily by intensive care nurses. When they perceived that society highly regarded the nursing profession and recognized nurses' expertise, this confirmed their feelings of pride based on their own convictions towards nursing and relevant expertise. In contrast, when they perceived that the public image of nursing was rather negative and the profession was diminished and not appreciated by society, this had the potential to impair their pride – especially when they felt that nursing was primarily associated with assisting patients with personal hygiene and toileting and that nursing tasks were perceived as inferior to physician tasks:

*N: The [nursing] profession itself makes me proud, it's something good that we're doing. It's just the conditions and the way society treats us that I no longer care about it and no longer feel very proud of it.*

*I: What conditions et cetera do you mean exactly?*

*N: (…) The lack of appreciation in society, "Nurse, oh, I couldn't do that," but at the same time, "What major things do you do, because the doctors do all the important things." (I13)*

While they acknowledged that as ICU nurses, they were generally more respected by other people outside health care than nurses working in other areas, such as a general ward or in long-term care, they regretted this being the case.

**Organizational factors:** The nurses often stated that organizational factors and work conditions negatively impacted their professional pride – most often because they perceived these factors as barriers to provide satisfactory care. They reported that they often have to set priorities that they consider to be wrong, for example when they have to support organizational processes, spend a lot of time on documentation and computer work at the expense of direct care, and have to accompany physicians’ morning rounds without actually being asked to provide their perspective on the patient situation (while putting their patient care behind to do so). They stated that they had to care for too many patients due to inadequate nurse-to-patient ratios on the one hand and unfilled positions, sick leaves, and long-term staff absences on the other hand. This makes it difficult for them to provide patient-centered care and achieve positive outcomes through thoughtful care and personal contact. This was reported to hinder them from experiencing situational pride, and sometimes even to negatively affect their stable professional pride – especially when they evaluated the delivered care as unsatisfactory and contradictory to their personal and professional values.

**Recognition and appreciation:** When nurses receive credit, for example, from patients, their relatives, physicians, colleagues, nurse managers, or in their relationships, this seems to contribute to their pride. The nurses in our sample reported positive workplace interactions – for example with patients and relatives – which increased their situational pride, which is based on their individual performance and achievements.

*I always found it very impressive when patients thanked me PERSONALLY. When they said, "Were you there?" And you say, "Yes, I was there when you were admitted and we resuscitated you." Then they're like, "Man, you saved my life, thank you very much." That makes you INCREDIBLY proud, that's a great thing. (I7)*

The nurses stated that they feel recognized by physicians when they acknowledge their expertise and respect their nursing work. For them, this is reflected in working at eye level and being perceived as equal healthcare team members, when, for example, no hierarchies are apparent and the nurses do not merely carry out instructions but are actively involved in matters of therapy management.

On the contrary, when appreciation is lacking or the nurses experience negative workplace interactions with patients, colleagues, and physicians, this can hinder them from feeling proud, e.g., if their expertise and their work are not recognized, if they are downgraded or if inconsistent ways of working in the team result in patient care goals not being achieved. They do not feel proud when patients behave disrespectfully towards them and expect them to fulfill their every wish or when they feel unfairly criticized or blamed by colleagues or physicians. It also impairs their pride if their nurse managers do not value them. The lack of recognition and appreciation can affect situational pride on the one hand, but also stable pride if it is continually absent.

*I used to be proud, and people would look at me respectfully and say: "Wow, that's great that you're doing that." But I can't feel like that anymore; today, I feel like a trash scavenger or a doormat. (I3)*

### 3.8 Nurses' evaluation of care and treatment episodes

The experience of professional pride seems to be strongly grounded in the nurses’ individual cognitive-affective evaluation processes. These, in turn, are based particularly on their attitudes towards and their understanding of the nursing profession. We found that the evaluation of actual care and treatment episodes plays a central role for the nurses' professional pride and that this evaluation includes a comparison of the delivered care with the nurses' ideals. These evaluation processes are implicit and explicit, and sometimes the nurses discuss them with their colleagues or in the interprofessional team. In these negotiation processes, the professional attitudes and values of the nurses, which contribute to their ideal of good care, especially their aspiration for person-centered care, play a crucial role. A positive match between the care and treatment provided and the nurse's ideals seems to promote situational pride. An opposing alignment may also occur, e.g., when the nurse succeeds in providing good patient care (in her own evaluation) only partially or not at all. This may lead the nurse to feel little or no situational pride in their work, while it seems to have little effect on their stable professional pride. However, there are situations when care is or must be provided in a manner that violates the nurse's ideals. Analyzing our data, we found that a cumulation of such situations resulted in negative emotions that seemed to have affected the nurses’ stable pride and, in certain instances, have raised doubts about maintaining the profession. The nurses reported to feel unable to live up to their professional values, and their everyday workplace performance no longer aligns with their original professional attitudes and beliefs. Therefore, a stable sense of pride is hardly possible for them anymore; their pride is rather fragile. The more the nurses' values and ideals are violated in these processes, the more extensive and far-reaching the negative emotional consequences seem to be for them.

In the nurses’ narrative accounts, we found minor and major aspects they considered to be unsatisfactory care, leading to minor or major consequences. For example, the nurses experience unsatisfactory care when they realize that they lack certain knowledge or skills or that they are unable to perform shift tasks due to a lack of time or can only complete them superficially. In such situations, negative emotional consequences for the nurses appear to be minimal if the patient's needs and the nurses' personal standards can be largely met. However, if there are significant differences, the nurses reported to experience this as distressing:

*You also have a high standard that you want to achieve. And if you don't achieve it, you are dissatisfied. And the more dissatisfied you are when you go home, the less VALUE you have for yourself. (I5)*

Particularly the nurses working in a general ward reported feelings of frustration due to changes in the care delivery compared to the past. They observed a deterioration of what they once perceived as good care and criticized healthcare for being increasingly oriented towards economic factors rather than being patient-centered. As patients’ needs that they sometimes cannot meet at all or in time, they cited attentiveness, caring behaviors, affection, and conversation, but also assisting in personal hygiene and sometimes even basic physiological needs such as assisting in toileting.

Ethical conflicts and a lack of involvement in treatment decisions were also identified as factors that make the nurses perceive care as unsatisfactory. They reported clinical situations in which they considered medical procedures not to be in the patients’ best interests, and felt burdened when these patients did not recover well afterwards or suffered due to side effects or follow-up treatments. In some cases, they criticized the medical clearance talks for not providing patients with a clear picture of the procedure's consequences and the postoperative period ("sugarcoating", I3). This perceived mismatch between patients’ needs and the actual treatment appeared to lead to a perception of unsatisfactory healthcare, which violates their ideals and thus impairs their pride.

Additionally, ICU nurses reported facing ethical conflicts when involved in high-risk procedures and treatments on patients they believe will not benefit. They criticized the lack of involvement in therapeutic decisions and decisions to limit or discontinue therapy.

*You can't decide. You have to do it, and you can't decide anything. You may express your opinion, for example. That's all you can do. (I1)*

### 3.9 Possible effects of nurses’ professional pride

Our data also contained initial hints regarding possible effects of nurses' professional pride. The nurses often reported the effects they perceived in connection with their definitions of professional pride. They stated that, for them, professional pride means enjoying their work and feeling deeply connected and in harmony with their profession. Professional pride also made them commit to the nursing profession in front of others:

*Professional pride is when I can say that I am proud to pursue this profession. For example, when someone asks me, "What do you do for a living?", and I say, "I'm a nurse, and I think it's great, and I'm proud of it." (I3)*

They associate professional pride with the intention of staying in the nursing profession while constantly developing personally and as a professional:

*To have chosen a profession, pursued a career, and to know that you want to continue doing that for several years. To work with a vision for the future, not just in the here and now, but that the development continues and that you also continue to evolve. But that you still stay in the same profession nonetheless. (I5)*

They also emphasized that their professional pride is a factor that determines their behavior and demeanor in the interprofessional team, in particular in their collaboration with physicians: They describe professional pride to be a driving force for their patient advocacy, for example when they speak up during rounds or question physicians' orders for the patient's benefit.

## 4 Discussion

This qualitative study investigated nurses’ professional pride, based on interviewing 17 nurses from a German university hospital. We found that the nurses experience professional pride in two ways: stable and situational professional pride. Individual cognitive-affective evaluation processes seem to play a crucial role in professional pride, being present in the three sources of professional pride which we identified in our data: (1) the nurses’ attitudes towards the profession, (2) the nurses’ knowledge and competencies, and (3) the nurses’ care activities and successes. Furthermore, we found three categories of external factors that influence whether or not the nurses can activate these sources and actually feel professional pride in the workplace or even in general: (1) societal factors, (2) organizational factors, and (3) workplace acknowledgement and appreciation. Of these, organizational factors seem to play a major role, as they often influence how patient care and treatment are delivered and perceived. For example, the impression of having to care for too many patients in too little time – given the relatively large patient-nurse ratio in Germany compared to other countries (Rafferty et al., 2019) –, combined with the perceived need to prioritize organizational processes over patient needs, often leads nurses to feel that the care they provide to their patients is suboptimal. In our study, we identified the nurses’ evaluation of care and treatment episodes to be crucial for their feelings of professional pride: When they rated the care as satisfactory or successful, they felt situational pride, whereas in episodes of unsatisfactory or unsuccessful pride, they did not. If, in the latter case, their ideals were hurt, this could also impair their stable pride and even lead them to doubt whether they can continue in the profession. Experiencing professional pride, on the other hand, made them enjoy and commit to their profession and was cited as a factor that positively influences their decision to stay in the profession. Additionally, they also cited their professional pride as a contributing factor that helped them stand up for their ideals in patient care – e.g. in the interprofessional team – and advocate for patients’ rights and interests.

Since there is currently no standard definition of nurses' professional pride, we propose the working definition provided in Box 1 as a summary of our findings and the findings from the literature. This can serve as a basis for further research on this phenomenon, while taking into account the limitations of our study.

Box 1 **Proposed definition for nurses’ professional pride**

Nurses’ professional pride is a positive emotion that is experienced both situationally and stably and goes beyond mere job satisfaction. It results from a complex interaction of nurses’ individual attitudes, beliefs and motivation towards the profession, their professional knowledge and competencies and care activities and achievements on the one hand and external influencing factors related to the societal valuing of the nursing profession, the organizational work conditions and the workplace acknowledgments on the other hand. Nurses’ attitudes, knowledge and competencies provide the framework for their individual assessment of self-perceived accomplishments which is central to their experience of situational professional pride and, in the long-term, for their stable pride: The more satisfactory the outcome of this iterative individual evaluation process is, the more likely nurses feel situational pride that is important to keep their professional pride stable over time.

Alt-text: Unlabelled box dummy alt text

### 4.1 Comparison of our findings with existing literature

In our study, we found aspects confirming findings from our preliminary literature search on factors assumed to influence nurses’ professional pride (Supplement 1). For example, the sources of nurses‘ pride – nurses‘ attitudes towards the profession, their knowledge and competencies as well as their direct care activities and successes, have also been addressed in the studies by Nunstedt et al. (2020), Roth et al. (2022), Sumner (2008) as well as Hølge-Hazelton and Berthelsen (2021). Self-assessment of one's own performance based on self-defined criteria is also mentioned in the literature (Byers, 1990; Combrinck, 2018). However, many of these studies did not have the phenomenon of professional pride in their focus or mentioned it only in passing. In contrast, we designed our study primarily to examine hospital nurses’ understanding of professional pride, their experiences with it, and the associated sources and contributing factors. As a result, we could develop an exploratory conceptual model that interconnects the types, sources, external influencing factors and mediating mechanisms of nurses’ professional pride. We concluded from our data that the nurses’ constant, implicit, or explicit evaluation process of care and treatment episodes seems to be central to the experience of situational and stable professional pride. When making these judgements, they seem to draw on the professional ethics of nursing, as formulated for example in the International Council of Nurses' Code of Ethics for Nurses (2021), as well as on their personal moral convictions.

In the meantime, another qualitative study on nurses’ professional pride has been conducted in Germany (Adlhoch, 2024a, 2024b). Adlhoch (2024a) interviewed 12 nurses from different settings (acute and long-term care, outpatient care, forensic care) and different regions in Germany via telephone and performed a qualitative content analysis on their interpretations of the phenomenon of nurses’ professional pride. Based on these data, she identified a “common core” of professional pride which was independent of the care setting or the length of professional experience: Different sources of professional pride, e.g. care achievements, self-efficacy, and direct appreciation, were categorized into eight “factors of professional pride”, namely (1) nursing expertise, (2) nursing and care values, (3) nursing performance, (4) positive self-assessment, (5) public self-presentation, (6) external recognition, (7) working conditions, and (8) identification with the profession. These findings support our analysis, as the researcher identified similar elements: We identified three sources of nurses’ professional pride (attitudes towards the profession, knowledge and competencies, care activities and successes), which seem to be similar to the sources (1) to (4) and (8) derived by Adlhoch. Additionally, we found societal factors, organizational factors, and workplace acknowledgment and recognition to be external influencing factors, which are similar to Adlhoch’s factors (5) to (7). Furthermore, Adlhoch published another paper where she identified nine causes or triggers of moments in which her interviewed nurses experienced pride (Adlhoch, 2024b): (1) Going the extra mile, (2) Being aware of one’s acquired nursing expertise, (3) Putting nursing values into practice, (4) Demonstrating capability to act (i.e. being able to act in challenging situations, e.g. emergencies), (5) Evaluating one’s own performance positively, (6) Performing nursing tasks professionally, (7) Receiving external recognition, (8) Achieving visible success through nursing care, and (9) Having a professional standing (i.e. actively working on eye levels with physicians). These aspects can also be found in our data, especially in the pride source *nurses’ care activities and successes*. Furthermore, they support our distinction between situational pride (e.g. 4, 5, 7, and 8) and stable pride (e.g. 2 and 9). Additionally, in line with our findings, Adlhoch’s data also indicate that nurses evaluate their performance as well as the care provided against a set of nursing and care values and that their professional pride involves both cognitive and emotional aspects (Adlhoch, 2024a, 2024b). Thus, Adlhoch’s findings support both our results and our proposed definition of nurses’ professional pride. However, Adlhoch does not present a conceptual model that connects her findings or indicates relationships among them. Furthermore, since she did not focus on a specific care setting, specific contextual factors and their influence are not discussed in detail.

A distinct difference of our results was found when compared to the findings in the study by Sneltvedt and Bondas (2016), in which nurses primarily viewed their close relationship with their care recipients as a source of their professional pride. This relational aspect only played a minor role in our study, which, however, could be due to the different setting: While the present study included nurses from acute inpatient care, Sneltvedt and Bondas (2016) examined nurses in outpatient or inpatient long-term care. In this setting, the relationship with care recipients is, on the one hand, significantly longer-lasting, and on the other hand, the focus of care is primarily on maintaining health and independence, whereas in the hospital, the primary focus is on the short-term improvement of an acute health problem. However, Adlhoch (2024 a, 2024b) also did not explicitly touch on the aspect of relationships, although she also interviewed nurses working in nursing homes and outpatient care. An explanation could be the nature of nursing in Germany, where a strong tasks-oriented approach to nursing care is still common (Kühme, 2020). Additionally, nurses in Adlhoch’s as well as our sample had rather long professional experience, with the biggest proportion working in nursing for more than ten years (our study: 82%, Adlhoch’s study: 67%), meaning that they were socialized with a rather functional focus of care (e.g., taking vital signs for the whole ward, while others were providing medication for all patients and others accompanying medical rounds) instead of taking responsibility for all care activities for a patients group in a shift. It can therefore be assumed that a patient-centered approach to nursing – in which building a relationship between nurse and patient is of crucial importance – still does not significantly shape their actions and ways of thinking today.

The socialization of nurses in Germany, where nursing and patient care – particularly in hospitals – remains highly physician-centered, could also explain why nurses in our study, as well as in that of Adlhoch, cited collaboration with physicians on eye level as a factor relevant to their feelings of pride. This aspect might be influenced by differences in nursing education: In contrast to many other countries, undergraduate nursing education is still dominated by vocational training in Germany (Bergjan et al., 2021), and therefore most nurses do not hold a university degree. Consequently, disparities in education between nurses and doctors can also contribute to hierarchies within the health care team.

### 4.2 Implications for research

Future research should assess the transferability of our results to nurses in other countries or working in other contexts. For example, it would be of interest to explore whether the elements and mechanisms we identified in our study differ in nurses who work in pediatric or psychiatric wards in hospitals, based on their different approach, scope of work and care recipients. Additionally, there might be differences in nurses working in nursing homes where care relationships are mostly long-term and where there is no physician dominance. There might also be differences in other health care systems where nurses generally have academic education, a more independent scope of practice and work in less physician-centered systems. More insights from research would contribute to a deeper understanding of the phenomenon and the development of a robust definition of nurses’ professional pride. Having a better understanding of the phenomenon will enable researchers to develop psychometrically robust instruments to assess or measure professional pride in bigger samples of nurses. These data can be used to study possible statistical correlations of professional pride and other phenomena, e.g. work satisfaction, intention to stay, patient-centered care competency or even specific care outcomes. Additionally, the effect of interventions targeting nurses’ professional pride (e.g. education, changes in the work environment, in team culture or in structures of interprofessional collaboration) can be assessed. Besides, a deeper understanding of the phenomenon could help to address professional pride, its development, and maintenance in nursing education, as attitudes, knowledge, and skills are a major focus in the professional training of nurses. Raising awareness of professional pride among future nurses and highlighting ways of achieving it could be beneficial, particularly given the existing hints that it may have an impact on nurse retention (Roth et al., 2022; Sneltvedt and Sørlie, 2012; Valizadeh et al., 2018).

## 5 Strengths and limitations

Based on the narrative accounts of the nurses in our research sample, who work in different wards and have different specializations and educational backgrounds, detailed descriptions of nurses’ professional pride could be analyzed in this study. On this basis, we were able to provide a comprehensive description of the phenomenon under study and formulate hypotheses regarding possible relationships and mechanisms of action, which we have presented in the form of a preliminary conceptual model. However, only nurses from a single university hospital in Germany participated in the study, and the majority of them had a relatively long professional experience of more than ten years, which may limit the generalizability of the results.

The limited time frame of our study made it impossible to conduct an entirely criterion-based sampling. Additionally, we could not formally assess saturation due to the methodology used for our study. In framework analysis, assessing data saturation by common measures such as using the stopping criterion after additional interviews have not revealed new findings or by code frequencies counts (Hennink and Kaiser, 2022) are not suitable. This is because the analysis of the interviews by grouping similar statements and looking for patterns does not begin until all data have been collected, and because there are no codes in a sense that their frequencies can be counted. The non-iterative approach regarding data collection and analysis also hinders assessing theoretical saturation (Rahimi and Khatooni, 2024). We furthermore do not claim meaning saturation (Rahimi and Khatooni, 2024) for our study. In later phases of the data collection process, however, we gained a deeper understanding which helped us to make better sense of the data collected earlier. The most striking irritation for us were the interview statements by two nurses (I8, I10) that contradicted our assumption that pride is felt in certain situations. This made us take a closer look at the temporal dimensions of the experience of pride and consequently led to our distinction between situational and stable pride. This distinction then proved helpful for differentiating the sources of pride in all interviews. Additionally, it emerged that certain patterns and correlations regarding our phenomenon of interest might exist, and we perceived our data as internally consistent. On the other hand, it cannot be ruled out that including additional nurses into the study would have yielded further insights. It must also be considered that we included only nurses working at one hospital in the study. Therefore, our findings should be interpreted with caution and viewed as hypothesis-generating insights that require further investigation.

The involvement of author 1 in clinical practice, i.e., ICU care, and her preconceptions were addressed with strategies to increase reflexivity (see Section 2.6). Neither in interview situations nor in reviewing data transcripts we observed signs of differences in interview conduct with nurses from ICUs or general wards. For example, there were no instances of suggestive questions being asked or specific statements being deliberately emphasized in the interviews with intensive care nurses. Furthermore, the interview statements could be analyzed without any gaps in the narratives being noticeable, due to a possible assumption of shared knowledge. The multi-professionalism of the authors enriched the subsequent data analysis.

## 6 Conclusions

In our qualitative study on the professional pride of nurses working in a German university hospital, we found that the nurses feel pride in different situations and for various reasons, both situationally and in a stable manner. We were also able to identify factors that promote and inhibit their feelings of pride. While their attitudes towards the nursing profession, their knowledge and competencies, and their care activities and successes appear to play an essential role in their experience if situational and stable pride, their evaluation of care and treatment episodes seems to plays the most crucial role: When rated as successful, this seems to maintain or increases their pride, while an evaluation as not satisfactory appears to have the potential to impair their professional pride, especially with accumulating negative evaluation outcomes over time. In our sample, particularly organizational factors lead to the care being considered unsatisfactory by the nurses. Understanding the sources and influencing factors of nurses' pride, and how to address them effectively in organizational settings, constitutes a field for further research.

## Data sharing statement

The participants in this study did not provide written consent for their data to be shared publicly, so, due to the sensitive nature of the research, supporting data is not available.

## Declaration of generative AI and AI-assisted technologies in the manuscript preparation process

During the preparation of this manuscript the authors used Grammarly to improve spelling and grammar. After using this tool, the authors reviewed and edited the content as needed and take full responsibility for the content of the published article.

## Funding sources

No external funding.

## CRediT authorship contribution statement

**Johanna Ristau:** Writing – review & editing, Writing – original draft, Visualization, Project administration, Methodology, Investigation, Formal analysis, Data curation, Conceptualization. **Roman Helbig:** Writing – review & editing, Formal analysis. **Angelika Schley:** Writing – review & editing, Investigation, Formal analysis, Data curation. **Patrick Ristau:** Writing – review & editing, Writing – original draft, Visualization, Formal analysis. **Corinna Peifer:** Writing – review & editing, Validation, Supervision, Conceptualization. **Katrin Balzer:** Writing – review & editing, Supervision, Resources, Methodology, Conceptualization.

## Declaration of competing interest

None.

## Appendix Supplementary materials

Image, application 1

Image, application 2

Image, application 3

Image, application 4

## Footnotes

Supplementary material associated with this article can be found, in the online version, at doi:10.1016/j.ijnsa.2026.100624.
