# Supplementary material for: How nurses in acute care experience professional pride: a qualitative study
Source: Int J Nurs Stud Adv. 2026 Jul 9;11:100624. doi: 10.1016/j.ijnsa.2026.100624 (PMC13427396; doi:10.1016/j.ijnsa.2026.100624)
Supplement: Supplementary file 1 [file mmc1.docx]

| **Study**  Country and setting | **Interest or aim of the study** | **Research approach** | **Addressing professional pride planned a priori** | **Key statements or findings concerning professional pride** |
| --- | --- | --- | --- | --- |
| **Aase et al. 2021**  Norway, Netherlands; inpatient long-term care and home care | Conceptualization of "quality" by nursing staff and managers in care facilities and outpatient care | qualitative | no | Professional pride as an essential quality dimension in healthcare; pride in the ability to provide high-quality care to every patient; pride is synonymous with being a good fellow human being |
| **Brenner et al. 2010**  USA; hospital | Documentation of caring activities: Preferred format and influence on nurses' awareness of caring dimensions | mixed  methods | no | Pride in caring behaviors, with caring being seen as the cornerstone of the nursing profession |
| **Byers 1990**  USA; hospital | Nurses' views on nursing practice and ward ethos, as well as observable nursing practice and ethos on the ward | mixed  methods | no | Pride in one's own work based on one's own assessment; leading in managing the care of one's patients; pride in statements from physicians regarding the completion of their instructions |
| **Chegini et al. 2019**  Iran; hospital | Relationship between occupational stress, work-related quality of life, and turnover intention among intensive care nurses | quantitative | yes (1 item in a questionnaire) | Professional pride being defined as satisfaction with the profession and the organization by the authors; professional pride as a protective factor regarding ambitions to change the profession |
| **Combrinck 2018, Combrinck et al. 2020**  South Africa; hospital | Factors influencing nurses' professional dignity | qualitative | no, but professional dignity | Pride in being a nurse and wearing the uniform; pride in the important role in patient care; pride in doing one's best in the job and solving problems, pride in maintaining good nursing standards and providing good patient care, which brings additional recognition from patients and respect from colleagues; pride in the level of professionalism of other nurses; pride in academic standing/academic achievements that contribute to being on eye level with physicians; vulnerability of pride through mistakes, inadequate resources and ethically challenging situations, which can lead to failure to complete tasks and to burnout; the presence of professional pride leads to a need to carry and uplift the profession as well as to stay in it and has a positive impact on professional dignity |
| **Hannawa et al. 2021**  Switzerland; hospital | Criteria applied by doctors, nurses, and patients when evaluating the quality of care situations as good or bad | mixed  methods | no | Pride resulting from extraordinary care successes and an indicator of high-quality patient care |
| **Hølge-Hazelton &  Berthelsen 2021**  Denmark; hospital | Nurses' experiences of practices in hospital departments with low nurse turnover rates concerning culture, work environment, nursing competencies, and nurse management | qualitative | no | Pride in the quality of their care and their expertise; pride in the good care on their ward and good collaboration with their competent colleagues; pride gave the nurses the strength to continue working in their profession |

| **Study**  Country and setting | **Interest or aim of the study** | **Research approach** | **Addressing professional pride planned a priori** | **Key statements or findings concerning professional pride** |
| --- | --- | --- | --- | --- |
| **Hov et al. 2009**  Norway; inpatient long-term care | Experience of end-of-life care by nurses in long-term inpatient care | qualitative | no | Pride in the official instruction to nurse a patient back to life or a peaceful death; pride in being close to the patients and possessing important knowledge about patients' needs; feelings of disrespect and inferiority in the event of criticism or noticeable hierarchies in one's nursing team or in contact with nurses in the hospital and physicians |
| **Nunstedt et al. 2020**  Sweden; hospital | Explore and describe factors explaining why hospital nurses remain in the workplace | qualitative | no | Pride of the nurses in their professional role and the responsibility it entails; pride in one's expertise; pride in the work carried out by oneself and in the team and its positive effects on patients; promoting professional pride can maintain the good health of nurses |
| **Nydahl et al. 2015**  Germany; hospital | Experience of work pride and recognition by intensive care nurses in their daily work | qualitative | yes (work pride) | Pride in building relationships with patients and being able to help them, in the individual care and caring behaviors towards patients and relatives; pride in own skills and competencies (patient observation, additional qualifications, specialist training, having knowledge and passing it on to others, ability to improvise, meeting requirements and mastering stressful situations), pride in improving patients' conditions and providing successful patient care as a team; pride in staying in the nursing profession for a long time; pride in recognition, praise and trust from doctors, patients, relatives, and managers; problems with pride due to lack of staff and time, ethical challenges and lack of recognition |
| **Nydahl et al. 2016**  Germany; hospital | Extent of work pride and experience of recognition among intensive care nurses | quantitative | yes (work pride) | Pride in improving the condition of patients and acting effectively as a team in emergencies; pride in performing mobilization; pride due to comfort of relatives in patients' dying process; pride being impaired by bad mood in the team and a focus on mistakes, failure to achieve goals, the occurrence of errors and a lack of time and personnel; a positive correlation between being involved in medical decisions and feeling proud |
| **Ramvi 2015**  Norway; hospital | Personal and professional experiences of a nurse influencing her self-image as well as the influence of this self-image on her professional practice | qualitative | no | No pride in the nursing profession as such, as it does not require any special knowledge or skills |
| **Roth et al. 2022**  Germany; hospital | Nurses' perceptions of push and pull factors to leave or stay in the profession | qualitative | no | Pride in the knowledge and the activities of the nursing profession; pride in the nursing profession as a socially relevant and important profession and a calling; professional pride has a positive impact on the image of the nursing profession and can, therefore, make the profession attractive to young people; professional pride as a retention factor |
| **Study**  Country and setting | **Interest or aim of the study** | **Research approach** | **Addressing professional pride planned a priori** | **Key statements or findings concerning professional pride** |
| **Sneltvedt & Sørlie 2012**  Norway; inpatient long-term care and home care | Challenges for managers and colleagues when newly qualified nurses start their first job in inpatient long-term care or outpatient care | qualitative | no | Pride after graduation; appreciation of professional pride by employers can increase nurses' quality of work and improve retention |
| **Sneltvedt & Bondas 2016**  Norway; inpatient long-term care and home care | Experience of professional pride by newly graduated nurses in inpatient long-term care or outpatient care | qualitative | yes | Professional pride as a complex phenomenon with relational, dynamic, and collective dimensions; relationship with patients as the most important source of pride; pride in doing the right thing or doing something good for people and being a good nurse despite little experience; lack of pride when failing to provide good care, pride in specialist knowledge; pride in high-quality patient care as a team; pride due to recognition and acknowledgment by patients, relatives, the nursing and interprofessional team; however, pride is diminished if recognition and acknowledgment are lacking |
| **Sumner 2008**  USA, UK and New Zealand; Hospital | Nurse/patient relationship in three countries | qualitative | no | Pride in the nursing profession itself and in the fact that it is sometimes very hard work; pride in nursing activities, caring behaviors, and commitment to patients; pride in specialist knowledge, judgment, and practical skills |
| **Valizadeh et al. 2016**  Iran; hospital | Threats to nurses' dignity and their intent to leave the profession | qualitative | no | Impeded pride in the nursing profession due to its intangible nature, physician dominance, and negative societal image of nurses; lack of professional pride compromises dignity and can lead to leaving the profession |
| **Vikström & Johansson 2019**  Sweden; inpatient long-term care | Nursing home staff's experiences of how a quality development project influenced their work | qualitative | yes | Pride in experience and competence gained through direct care work; pride in doing good work for the benefit of residents, based on own professional judgment |

These findings were sorted into seven initial themes, which formed sensitizing concepts for the analysis of our interview material: (1) The nursing profession itself, (2) Nursing quality, commitment, performance, and success, (3) Competencies, specialist knowledge, and skills, (4) Direct and individual care with responsibility and humanity, (5) Overcoming challenges and solving problems, (6) Receiving appreciation and recognition and
(7) Working in a team.

**References**

Aase, I., Ree, E., Johannessen, T., Strømme, T., Ullebust, B., Holen-Rabbersvik, E., Thomsen, L. H., Schibevaag, L., van de Bovenkamp, H. & Wiig, S. (2021). Talking about quality: how 'quality' is conceptualized in nursing homes and homecare. *BMC Health Services Research*, *21*(1), 1–12. <https://doi.org/10.1186/s12913-021-06104-0>

Brenner, Z. R., Dimitroff, L. J. & Nichols, L. W. (2010). Documentation of nursing care behaviors. International Journal for Human Caring, 14(4), 7–13. <https://doi.org/10.20467/1091-5710.14.4.7>

Byers, S. R. (1990). Relationships among staff nurses' beliefs, nursing practice and unit ethos (PH.D.). Ohio State University.

Chegini, Z., Asghari Jafarabadi, M. & Kakemam, E. (2019). Occupational stress, quality of working life and turnover intention amongst nurses. Nursing in Critical Care, 24(5), 283–289. <https://doi.org/10.1111/nicc.12419>

Combrinck, Y. (2018). Strategies to preserve the professional dignity of nurses in a demanding healthcare environment. University of Pretoria, Pretoria. <https://repository.up.ac.za/handle/2263/67981>

Combrinck, Y., van Wyk, N. C. & Mogale, R. S. (2020). Nurses' professional dignity in private health care: a descriptive phenomenological study. *International Nursing Review*, *67*(3), 395–402. <https://doi.org/10.1111/inr.12602>

Hannawa, A. F., Wu, A. W., Kolyada, A., Potemkina, A. & Donaldson, L. J. (2021). The aspects of healthcare quality that are important to health professionals and patients: A qualitative study. *Patient Education and Counseling*, 1–10. <https://doi.org/10.1016/j.pec.2021.10.016>

Hølge-Hazelton, B. & Berthelsen, C. B. (2021). Why nurses stay in departments with low turnover: A constructivist approach. *Nordic Journal of Nursing Research*, *41*(3), 158–165. <https://doi.org/10.1177/2057158521991434>

Hov, R., Athlin, E. & Hedelin, B. (2009). Being a nurse in nursing home for patients on the edge of life. *Scandinavian Journal of Caring Sciences*, *23*(4), 651–659. <https://doi.org/10.1111/j.1471-6712.2008.00656.x>

Nunstedt, H., Eriksson, M., Obeid, A., Hillström, L., Truong, A. & Pennbrant, S. (2020). Salutary factors and hospital work environments: a qualitative descriptive study of nurses in Sweden. *BMC Nursing*, *19*(1), 125. <https://doi.org/10.1186/s12912-020-00521-y>

Nydahl, P., Hähnel, A. & Hermes, C. (2015). "Das macht mich dann stolz": Studie zum Arbeitsstolz in der Intensivpflege. *Die Schwester - Der Pfleger*, *54*(5), 86–91. <https://www.bibliomed-pflege.de/sp/artikel/24090-das-macht-mich-dann-stolz>

Nydahl, P., Krotsetis, S., Hähnel, A. & Hermes, C. (2016). Pflegestolz und Wertschätzung in der Intensivpflege: Nursing pride and acknowledgement in critical care. *Pflegezeitschrift*, *4*(69), 1–6.

Ramvi, E. (2015). I am only a nurse: a biographical narrative study of a nurse's self-understanding and its implication for practice. *BMC Nursing*, *14*(1), 1–9. <https://doi.org/10.1186/s12912-015-0073-y>

Roth, C., Wensing, M., Breckner, A., Mahler, C., Krug, K. & Berger, S. (2022). Keeping nurses in nursing: a qualitative study of German nurses' perceptions of push and pull factors to leave or stay in the profession. *BMC Nursing*, *21*(1), 1–11. <https://doi.org/10.1186/s12912-022-00822-4>

Sneltvedt, T. & Sørlie, V. (2012). Valuing Professional Pride and Compensating for Lack of Experience: Challenges for Leaders and Colleagues Based on Recently Graduated Nurses' Narratives. *Home Health Care Management & Practice*, *24*(1), 13–20. <https://doi.org/10.1177/1084822311412592>

Sneltvedt, T. & Bondas, T. (2016). Proud to be a nurse? Recently graduated nurses' experiences in municipal health care settings. *Scandinavian Journal of Caring Sciences*, *30*(3), 557–564. <https://doi.org/10.1111/scs.12278>

Sumner, J. (2008). Is caring in nursing an impossible ideal for today's practicing nurse. *Nursing Administration Quarterly*, *32*(2), 92–101. <https://doi.org/10.1097/01.NAQ.0000314537.98746.00>

Valizadeh, L., Zamanzadeh, V., Habibzadeh, H., Alilu, L., Gillespie, M. & Shakibi, A. (2018). Threats to nurses' dignity and intent to leave the profession. *Nursing Ethics*, *25*(4), 520–531. <https://doi.org/10.1177/0969733016654318>

Vikström, S. & Johansson, K. (2019). Professional pride: A qualitative descriptive study of nursing home staff's experiences of how a quality development project influenced their work. *Journal of Clinical Nursing*, *28*(15-16), 2760–2768. <https://doi.org/10.1111/jocn.14884>
