# Supplementary material for: How nurses in acute care experience professional pride: a qualitative study
Source: Int J Nurs Stud Adv. 2026 Jul 9;11:100624. doi: 10.1016/j.ijnsa.2026.100624 (PMC13427396; doi:10.1016/j.ijnsa.2026.100624)
Supplement: Supplementary file 2 [file mmc2.docx]

| **Subject area** | **Main and follow-up questions** |
| --- | --- |
| **Participant's degree of professional pride** | - How proud are you to work as a nurse?   - In which situations in your day-to-day work do you feel proud to work as a nurse?     - Please provide one or two examples from your last work week! |
| **Definition of professional pride** | - We have already heard a lot about professional pride. Once again, in a nutshell: How would you define professional pride? |
| **Supporting  and inhibiting factors** | - Which factors or situations make it easier and which make it more difficult for you to be proud of your profession? - What would have to change for you to feel (even) more pride in your daily nursing work?   *If not yet discussed by then:*   - To what extent do you receive recognition for your work as a nurse?   - By whom (patients, relatives, other professional groups in the hospital, personal environment, society)?   - How is this recognition expressed? |
| **Expertise/ professional requirements** | - To what extent are you proud of your specialist knowledge and your special skills as a nurse? - To what extent do you need specialist nursing or medical knowledge to be a good nurse? - How important is it for you personally to maintain high standards and/or a high level of quality in your everyday nursing work? - In your opinion, which nursing skills are particularly important for high quality in your work?   - *Possible question:* What do you think of the statement "Anyone can do nursing care"? - What nursing and care outcomes are you (particularly) proud of in your everyday nursing work? - How important is it to you personally to keep your knowledge and skills up to date? |
| **Development opportunities/ prospects** | - From your perspective: What development opportunities in nursing contribute or could contribute to your professional pride? - What does "working at the bedside" mean to you?   - *Possible question:* To what extent is this something to be proud of? - In a perfect world, how and where would you like to work as a nurse?   - Which duties would you like to be responsible for?   - How much time would you like to work?   - What other working conditions would be important to you (e.g., work processes, working hours, team structure)? |

**Closing**: Is there anything else you want to say on this topic that we haven't discussed yet?

**Thanks and goodbyes.**

^1^Translated from German.
