# Supplementary material for: How nurses in acute care experience professional pride: a qualitative study
Source: Int J Nurs Stud Adv. 2026 Jul 9;11:100624. doi: 10.1016/j.ijnsa.2026.100624 (PMC13427396; doi:10.1016/j.ijnsa.2026.100624)
Supplement: Supplementary file 3 [file mmc3.docx]

**Data analysis: example of detected elements in summaries and grouped detected elements included in a category “**

This supplement gives an example of how we developed categories and categories: In interview summaries, we identified elements which were later pooled and sorted. We have used colors for the elements we identified in three selected summaries to make it visible where these elements appear in the subcategories that were then further developed to the category “Making a positive difference through your own contribution”. Detected elements from the summaries that are not in colored fonts were added to another subcategory or category; uncolored detected elements in the subcategories come from other interviews or interview passages.

**Summary 1: I1**

He feels proud when he does something good for patients. When he achieves that, he feels proud. When he does something good for patients so that they become healthier and feel better, allowing them to return to their homes or to their normal lives. He feels proud when he does something good for patients and has achieved something when they benefit from it. Then, when he goes home, he knows that he has done something good for someone, and that makes him proud.

**Detected elements:**

- Doing something good for the patient
- Achieved something positive for the patient/the patient benefits from the nurses' actions
- The patient becomes healthier as a result of the nurse's efforts
- The patient feels well due to the actions of the nurses
- Patient returns to normal life
- Feeling proud when going home

**Summary 2: I7**

Pride in the patient’s progress. Pride when the patient and their family feel comforted.

Situation: The patient had difficulty mobilizing and needed a lot of assistance. Two days later, you return and see the success of your work: he can already do it almost on his own, and he’s happy that he’s managing on his own. That makes you incredibly proud.

Pride due to gratitude from the patient and their family, whether through direct feedback or in letters. When a patient personally thanks you – for example, after resuscitation, for saving their life – that makes you incredibly proud. It means you’ve done your job well and made it possible for this person to continue living their life.

Pride in being able to solve problems; for example, finding a good, specific positioning for a patient, passing that on to the team, and making the patient happier or possibly less in pain as a result.

**Detected elements:**

- Patient’s progress
- Patient and family members feel comforted
- [Patient who has difficulty moving and requires a lot of guidance] Success that the patient can almost perform mobilization on his own
- The patient’s happiness with his progress in mobilization
- Expressions of gratitude from the patient and family members, either in person or by letter
- Personal thanks from the patient, e.g., after resuscitation, for saving his life
- Having done one’s job well and enabled a person to continue living
- When she is able to solve problems […]
- Finding a specific positioning and sharing it with the team so that the patient experiences less pain and feels happier

**Summary 3: I13**

When, as a team, you manage to save patients you had already given up on; when, over the course of several shifts, your individual efforts have prevented a (re)intubation. When you’ve taken over a critically ill patient and, through your work, ensure that they are no longer in a critical condition by the end of your shift. When you can help ensure that the patient is able to leave the ICU quickly. It makes you feel proud when you’ve had a good shift, had time for the patient, and were able to do your job effectively.

**Detected elements:**

- As a team, save patients who had already been given up on
- [To avoid (re-)intubation]/ Saving the patient from harm
- The patient’s health becomes more stable/is no longer critical as a result of the nurse’s work
- The patient is able to leave the ICU quickly thanks to the nurses’ efforts
- Having had a good shift, during which you had time for the patient and were able to carry out your duties, makes you feel proud

**Category: Making a positive difference through your own contribution**

**Positively influence the patient's state and condition**

- Achieved something positive for the patient/the patient benefits from the nurses' actions
- The patient becomes healthier as a result of the nurse's efforts
- Nurses’ ability to avoid (re)intubations
- Improving the patient’s respiratory status
- Achieving successes by adjusting the ventilator settings
- When a patient survives resuscitation without complications
- Making a neglected person look like a human being again through basic care is satisfying and a source of professional pride
- Having made a difference through one’s work/having helped the patient progress through one’s work
- Finding a specific positioning and sharing it with the team so that the patient experiences less pain and feels happier
- Working with medical technology such as ventilators directly ensures the patient’s life
- Improvements in mobility when the nurse has helped or advised the patient
- When you are partly responsible for the patient’s recovery because they are able to carry out their mobilization after you’ve explained the benefits
- The patient’s health becomes more stable/is no longer critical as a result of the nurse’s work
- The patient is able to leave the ICU quickly thanks to the nurses’ efforts
- Improving specific aspects

**Achieve visible/** **tangible results through nursing care**

- Making a neglected person look like a human being again through basic care is satisfying and a source of professional pride
- Progress in mobility, because it is noticeable
- The patient is able to leave the ICU quickly thanks to the nurses’ efforts
- Seeing how wounds heal and get better

**Improving the patient's comfort and well-being through nursing care**

- The patient feels well due to the actions of the nurses
- Thoughtful, well-planned nursing care that positively impacts the patient’s mental state and makes them happier
- Patient and family members feel comforted
- The patient’s happiness with his progress in mobilization

**Patient autonomy**

- Patient returns to normal life
- Success that the patient can almost perform mobilization on his own
- The patient’s happiness with his progress in mobilization

**Protect patients**

- [Has prevent tracheotomy in a patient]/Saving the patient from harm
- [To avoid (re-)intubation]/ Saving the patient from harm

**Participation/involvement in decision-making regarding the treatment process**

- Being involved in shaping the treatment and recovery process
