# Supplementary material for: How nurses in acute care experience professional pride: a qualitative study
Source: Int J Nurs Stud Adv. 2026 Jul 9;11:100624. doi: 10.1016/j.ijnsa.2026.100624 (PMC13427396; doi:10.1016/j.ijnsa.2026.100624)

**Initial visualizations of our data**

**Preliminary Model 1**: Sources of nurses’ professional pride. Own illustration.


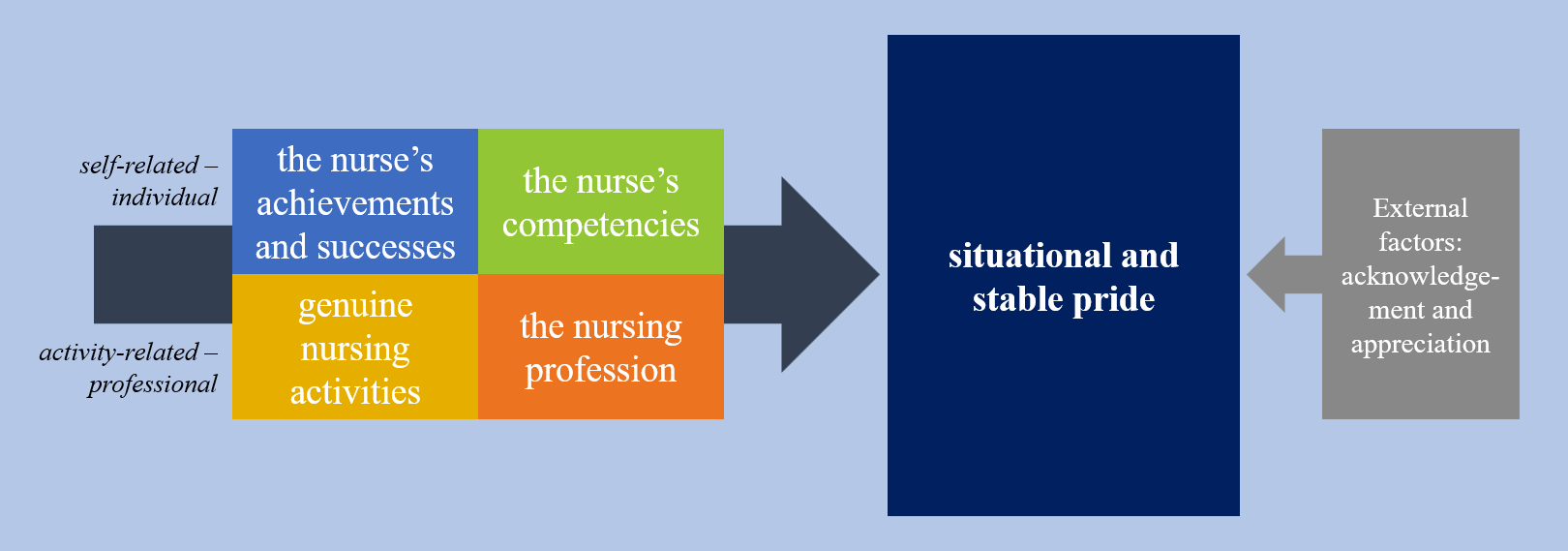


**Preliminary Model 2**: Factors inhibiting feelings of pride. Own illustration.


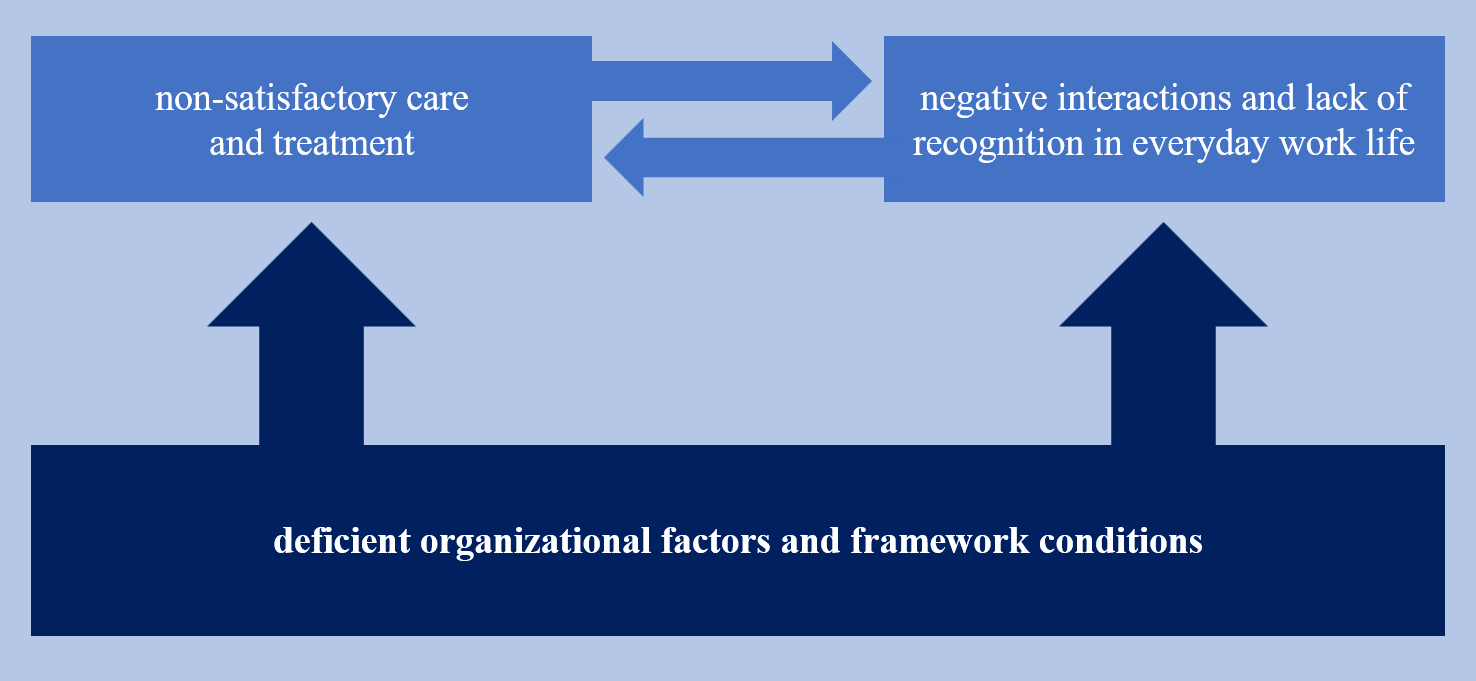


**Preliminary Model 3**: Nurses’ evaluation of care and treatment episodes. Own illustration.


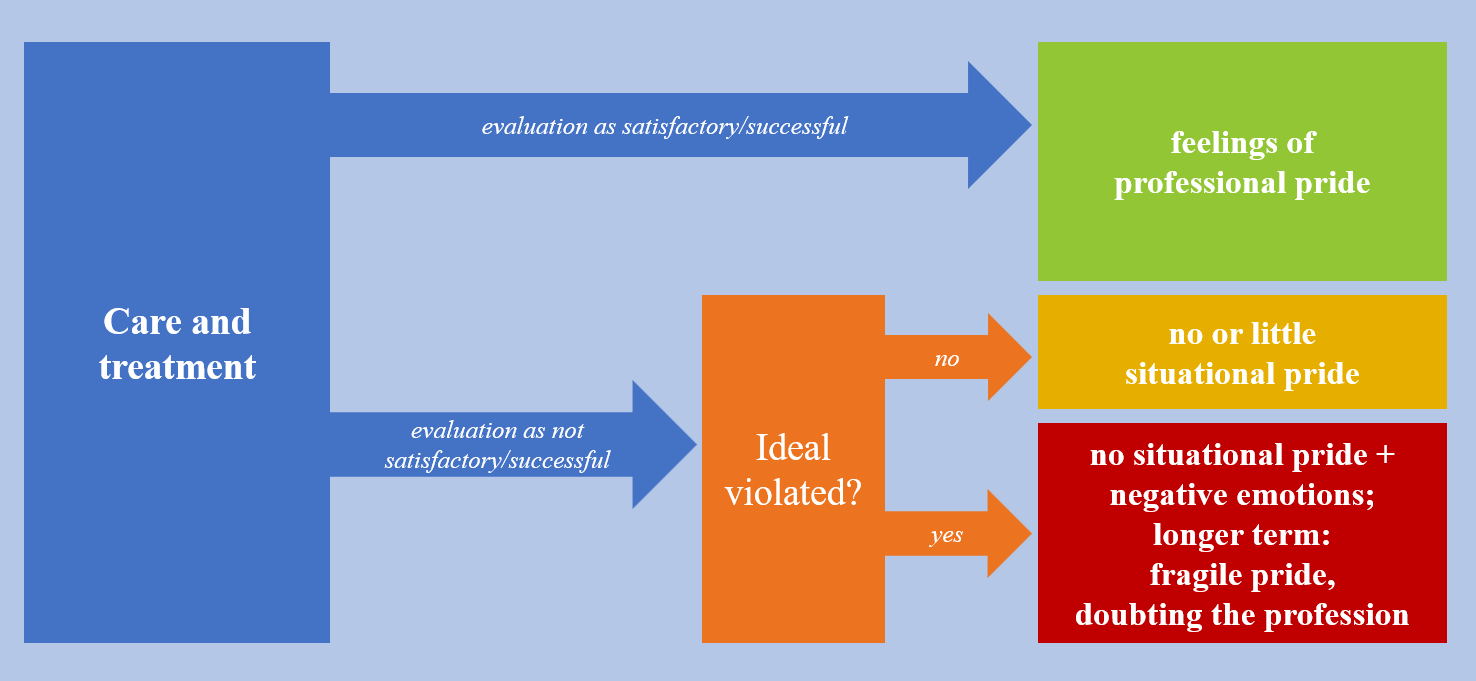

Supplement: Supplementary file 4 [file mmc4.docx]
